# Effects of Isochoric Freezing Conditions on Cut Potato Quality

**DOI:** 10.3390/foods10050974

**Published:** 2021-04-29

**Authors:** Yuanheng Zhao, Cristina Bilbao-Sainz, Delilah Wood, Bor-Sen Chiou, Matthew J. Powell-Palm, Liubiao Chen, Tara McHugh, Boris Rubinsky

**Affiliations:** 1 Technical Institute of Physics and Chemistry, Chinese Academy of Sciences, Beijing 100190, China; zhaoyuanheng16@mails.ucas.ac.cn (Y.Z.); chenliubiao@mail.ipc.ac.cn (L.C.); 2 Western Regional Research Center, U.S. Department of Agriculture, Albany, CA 94710, USA; de.wood@usda.gov (D.W.); bor-sen.chiou@usda.gov (B.-S.C.); tara.mchugh@usda.gov (T.M.); 3 Department of Mechanical Engineering, University of California at Berkeley, Berkeley, CA 94720, USA; mpowellp@berkeley.edu (M.J.P.-P.); rubinsky@berkeley.edu (B.R.)

**Keywords:** potato, isochoric freezing, texture, browning

## Abstract

Isochoric freezing is a pressure freezing technique that could be used to retain the beneficial effects of food storage at temperatures below their freezing point without ice damage. In this study, potato cylinders were frozen in an isochoric system and examined using full factorial combinations of three processing procedures (immersed in water, vacuum-packed and immersed in ascorbic acid solution), four freezing temperatures/pressures (−3 °C/37 MPa, −6 °C/71 MPa, −9 °C/101 MPa and −15 °C/156 MPa) and two average compression rates (less than 0.02 and more than 0.16 MPa/s). The effects of process variables on critical quality attributes of frozen potatoes after thawing were investigated, including mass change, volume change, water holding capacity, color and texture. Processing procedure and freezing temperature/pressure were found to be highly significant factors, whereas the significance of the compression rate was lower. For the processing procedures, immersion in an isotonic solution of 5% ascorbic acid best preserved quality attributes. At the highest pressure level of 156 MPa and low compression rate of 0.02 MPa/s, potato samples immersed in ascorbic acid retained their color, 98.5% mass and 84% elasticity modulus value. These samples also showed a 1% increase in volume and 13% increase in maximum stress due to pressure-induced hardening.

## 1. Introduction

Minimally processed products are one of the major growing segments in the food industry. However, fresh-cut fruits and vegetables have limited shelf life due to excessive tissue softening and cut surface browning. Preservation of food products at temperatures below their freezing point have been effectively utilized to decrease the rate of deterioration and extend the shelf-life. However, consumers perceive fresh produce as healthier, fresher and higher quality than pre-packaged and frozen produce [1].

Pressure-related freezing techniques have attracted a lot of interest for increasing freezing efficiency and ensuring better frozen food quality [2]. These freezing technologies are based on the effects of pressure on phase transitions of water. The phase diagram of water shows that pressure decreases the phase change temperature of water from 0 °C at 0.1 MPa to −21 °C at 210 MPa as well as enabling the formation of various high-density forms of ice [3]. This phenomenon has led to the development of different high-pressure freezing processes for the preservation of foods, including pressure-assisted freezing, pressure shift freezing, freezing to ice III and low-temperature non-frozen storage under pressure. Previous reviews and studies had discussed the application of high-pressure freezing to food technology [4,5,6]. The authors had identified the advantages of high-pressure treatments in food freezing processes.

Isochoric freezing is also considered a high-pressure freezing technique that allows low-temperature storage without ice formation. During isochoric freezing, pressure is achieved by reducing the temperature of an aqueous system in a rigid and constant volume chamber. Under these conditions, in which the volume is constrained, the formed ice expands to generate hydrostatic pressure inside the rigid chamber. This pressure then depresses the freezing point of the aqueous solution and ice will form until the effective freezing point of the system is equal to the surrounding temperature. At this point, there are two phases coexisting at a constant subfreezing temperature: a solid ice phase and a liquid phase. Food products can be stored at subfreezing temperatures without any internal ice formation if the food products remain in the space occupied by the liquid phase during freezing. The potential harmful effects of pressure are minimized since the process path during cooling under isochoric conditions always follows the liquidus curve of the temperature-pressure phase diagram until reaching the triple point between ice I, ice III and liquid [7].

Recently, several papers had focused on the energy consumption during isochoric freezing [8], the effects of isochoric freezing on microbial inactivation [9,10] and the effects of isochoric freezing on food quality [11,12]. These studies showed the potential of isochoric freezing to reduce energy consumption during frozen storage, to inactivate pathogenic microorganisms and to preserve frozen foods’ physicochemical and nutritional properties.

Minimally processed potatoes offer the catering industry and households a simplified way of cooking. However, a peeled and cut tuber has a very short shelf-life of 5–7 days at 4–5 °C due to physiological ageing, biochemical changes and microbial spoilage [13,14]. In comparison, a potato has a shelf-life of 3–5 weeks at room temperature and 3–4 months in the refrigerator. The effects of isochoric freezing on quality aspects of minimally processed potatoes had been previously investigated [15,16]. The authors reported that cut potatoes frozen in an isochoric system showed better quality properties in terms of drip loss, volume shrinkage and texture than cut potatoes frozen at atmospheric pressure or individually quick-frozen potatoes. In this study, we examined the effects of isochoric freezing conditions (procedure, temperature/pressure and compression rate) on the quality properties (mass change, volume change, water holding capacity, color and texture) of cut potatoes after thawing. We used full factorial combinations of three processing procedures, four freezing temperatures/pressures and two compression rates.

## 2. Materials and Methods

### 2.1. Plant Material

Potato tubers grown in the Columbia Basin, WA (S. tuberosum L. of the “Russet Burbank” variety), were kindly provided by Lamb Weston (Richland, WA, USA). On arrival, potato tubers were stored at 5 °C for no more than 3 weeks. Potatoes were peeled and cut into cylinders (18 mm in diameter and 15 mm in height). The initial volume and weight were recorded.

### 2.2. Isochoric System

Two different pressure chambers were used in the isochoric experiments. The first chamber was obtained from HighPressure Equipment Company (Erie, PA, USA). The chamber was made of 4340 alloy steel. It had an inner diameter of 2.54 cm and an outer diameter of 8.13 cm. The total volume capacity was 66 mL. The second chamber was obtained from BioChoric LLC (Berkeley, CA, USA). This chamber was made of grade 5 titanium. It had an inner diameter of 2.54 cm and an outer diameter of 4.45 cm. The total volume capacity was 75 mL. The first chamber was used for the experiments performed at −3 °C/37 MPa, −6 °C/71 MPa and −9 °C/101 MPa, and the second chamber was used for the experiments performed at −15 °C/156 MPa. The pressure was monitored using an electronic pressure transducer (Stork Solutions Ltd., Hampshire, UK) connected to the chamber. The system was cooled using a recirculating bath (VWR AP 15R-40, Radnor, PA, USA) filled with a water and ethylene glycol (50:50) solution.

### 2.3. Experimental Protocol

Isochoric freezing requires the absence of air inside the chamber because of the high compressibility of air. Therefore, potatoes were processed following three different procedures. In the first procedure (P1), 8 potato cylinders from 3 different potatoes were directly placed in the isochoric chamber filled with distilled water. In the second procedure (P2), 8 potato cylinders from 3 different potatoes were vacuum-packed in a moisture-impermeable plastic bag (FoodSaver from Newell Brands, Hoboken, NJ, USA) using the FoodSaver vacuum sealer (Newell Brands, Hoboken, NJ, USA) and the bag was placed in the pressure unit filled with distilled water. In the third procedure (P3), 8 potato cylinders from 3 different potatoes were packed and immersed in an isotonic solution containing 5% ascorbic acid (AA) aqueous solution using a moisture-impermeable plastic bag (FoodSaver from Newell Brands, Hoboken, NJ, USA). The bag was then sealed and placed in the pressure unit filled with distilled water.

For the isochoric freezing treatment, the isochoric chamber was completely immersed in an insulated container connected to a recirculating cooling bath. The freezing temperature/pressure were set at −3 °C/37 MPa, −6 °C/71 MPa, −9 °C/101 MPa or −15 °C/156 MPa. The average compression rates were less than 0.02 MPa/s or more than 0.16 MPa/s by controlling the temperature of the bath. The samples were processed for 2 h at the selected conditions. After this, the pressure in the chamber was released by thawing the chamber in a water bath at room temperature for one hour before testing.

### 2.4. Mass Change, Volume Change and Water Holding Capacity (WHC) Determination

Sample dimensions and masses were measured before freezing and after thawing for at least four cylinders. Mass change was calculated gravimetrically and reported as the percent change in sample mass based on its initial mass. Volume change was determined using a digital caliper micrometer and reported as the percent change in sample volume based on its initial volume. Mass change and volume change were determined four times for each freezing condition.

Water holding capacity was performed applying low-speed centrifugation [17]. A thawed potato cylinder was placed in a 45 mL plastic centrifuge tube suspended on a perforated support. The sample was centrifuged at 10,000 rpm for 10 min. Measurements were done at 4 °C. Water holding capacity was measured as the percentage difference between the weight of the sample after thawing (*m*_1_) and the weight of the sample after centrifugation (*m*_2_) according to the following equation:

(1) $$\%\mathrm{WHC}=\frac{m_{1}-m_{2}}{m_{1}}\times 100$$

WHC was analyzed in triplicate using 3 potato cylinders from 3 different tubers.

### 2.5. Potato Color

The frozen samples were thawed for 1 h before performing color measurements to allow the occurrence of browning. A spectrophotometer (CM508D, Konica-Minolta Inc., Ramsey, NJ, USA) with an 8 mm CM-A196 Target Mask was used to analyze the surface color of six potato cylinders per freezing treatment. CIE (L*, a*, b*) values were recorded and browning index (BI) was determined using the following equation:

(2) $$\mathrm{BI}=\left[100\left(\frac{a+1.75L^{*}}{\left(5.645L^{*}+{\;a}^{*}-3.012b^{*}\right)}-0.31\right)\right]/0.172$$

where L* is the lightness, a* is the green-red color axis and b* is the blue-yellow axis.

### 2.6. Mechanical Properties

A Texture Analyzer (Stable Microsystems Ltd., TA-XT2i, UK) with a 50 mm diameter circular flat plate (TA-25 probe) was used to determine the mechanical properties of the potato samples following the procedure described in Luscher et al. [18], with minor modifications as described in Bilbao-Sainz et al. [15]. The samples were compressed to 50% deformation in a single compression-decompression cycle at a speed of 0.1 mm/s. Five randomly selected cylinders from three different tubers were tested for each freezing treatment.

### 2.7. Statistical Analysis

A multilevel full factorial design was applied to evaluate the effects of two numerical factors and one categorical factor on quality attributes of thawed potatoes frozen under isochoric conditions. The numerical factors were temperature (T) at four levels (−3, −6, −9 and −15 °C) and average compression rate (CR) at two levels (less than 0.02 MPa/s and more than 0.16 MPa/s). The categorical factor was the procedure (P) at three levels (samples immersed in water, vacuum-packed samples and samples packed in isotonic solution of 5% ascorbic acid aqueous solution). The ranges of the independent parameters were determined from preliminary experiments. A total of 24 experiments with 8 experiments for each level of categorical factor were performed. Minitab^®^ 19 software was utilized for the experimental design and to perform a statistical analysis of variance (ANOVA) on the individual and interactive effects of the factors. A *p*-value less than 0.05 was considered as statistically significant.

## 3. Results and Discussion

### 3.1. Effects of Isochoric Freezing Conditions on Mass Changes

**Figure 1:**
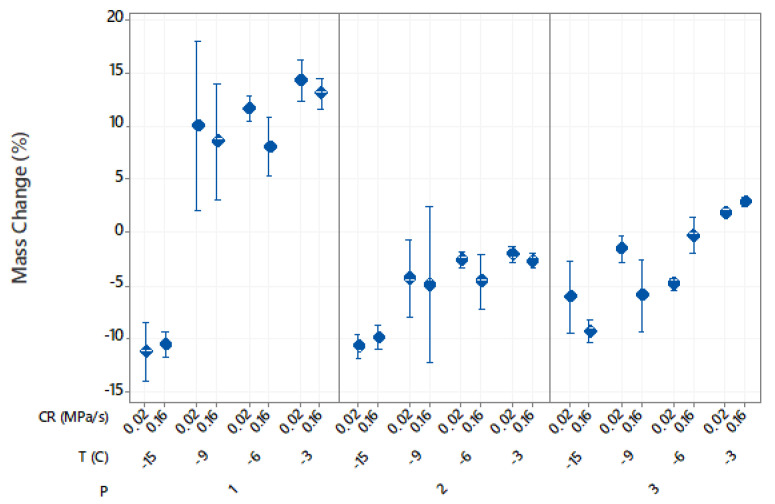
Effects of the isochoric freezing procedures (P), freezing temperatures (T) and compression rates (CR) on mass change of cut potatoes after thawing. The pressures were 37 MPa at −3 °C, 71 MPa at −6 °C, 101 MPa at −9 °C and 156 MPa at −15 °C. Data represent mean and 95% confidence interval for the mean.

Figure 1 shows the effects of the different freezing conditions on mass change of thawed potato samples. Mass changes ranged from 14% increase to 11% decrease depending on the isochoric freezing conditions. The ANOVA analysis of the factorial regression model (R2 = 96.65%) showed that the freezing temperature (F3,88 = 392.98, *p* < 0.001) and the processing procedure (F2,88 = 379.06, *p* < 0.001) had the greatest effects on mass changes. In comparison, the significance of the compression rate was much lower (Table 1).

The samples immersed in water and frozen at −3 °C, −6 °C and −9 °C showed increases in mass. Mass gain decreased from 13.6% at −3 °C to 9.2% at −9 °C. At the lowest freezing temperature of −15 °C, the potato sample lost 10.9% mass. The increase in mass might be due to water uptake in response to the difference in osmotic potential between the potato cells and the surrounding medium. Pressure can induce opposing effects on the mass balance. It can accelerate infusion through changes in cell permeability [19,20] but also allows the release of liquids [20,21]. Thus, the changes in mass with temperature/pressure were due to the balance of these two effects. The vacuum-packed samples lost more mass at −15 °C (10.3%) than at −3 °C (3.4%), indicating a progressive increase in the number of damaged cells at higher pressures. Additionally, the AA-immersed sample at the highest freezing temperature of −3 °C gained mass. However, the increase in mass was significantly lower for these samples (2.3%) than for water-immersed potatoes (13.6%), since potato samples were in osmotic equilibrium with the AA solution. A previous study found no changes in the cellular tissue of potatoes frozen at −3 °C under isochoric conditions, suggesting that the slight increase in mass was probably due to the pressure-induced impregnation of the intercellular spaces in the potato tissue with the external solution [15]. This effect highlights isochoric freezing as an interesting tool for nutrient diffusion into the intercellular spaces and pores of solid foods. The AA-immersed sample showed a slight mass loss of 3.7% at −9 °C and a 7.7% mass loss at −15 °C. Moreover, the compression rate had only a significant effect for AA-immersed samples, with mass losses being about 4% smaller at the lower compression rate. This indicated that the cellular structure was better preserved when the pressure was applied slowly.

### 3.2. Effects of Isochoric Freezing Conditions on Volume Changes

**Figure 2:**
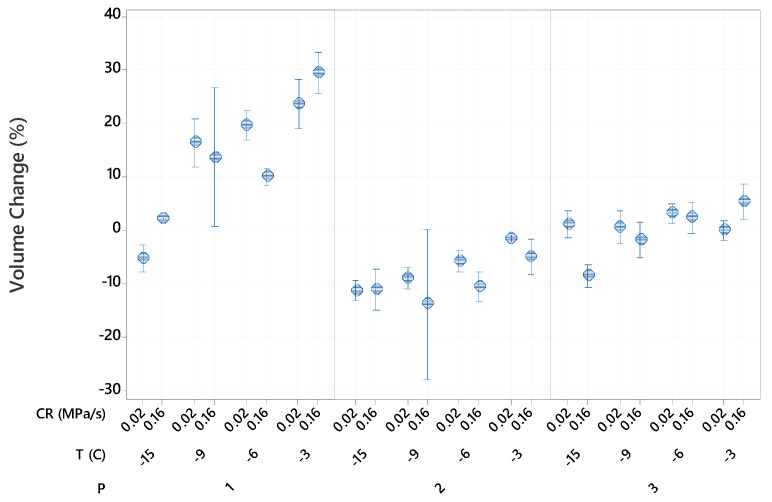
Effects of the freezing procedures (P), freezing temperatures (T) and compression rates (CR) on volume change of cut potatoes after thawing. The pressures were 37 MPa at −3 °C, 71 MPa at −6 °C, 101MPa at −9 °C and 156 MPa at −15 °C. Data represent mean and 95% confidence interval for the mean.

The volume changes ranged from 30% increase to 14% decrease, as shown in Figure 2. The ANOVA analysis of the factorial regression model (R2 = 96.61) showed that the processing procedure was the factor with the greatest effect on volume changes (F2,80 = 719.62, *p* < 0.001), followed by the freezing temperature (F3,80 = 169.77, *p* < 0.001). The effect of the compression rate on the changes in volume was significant, but lower (F1,80 = 11.95, *p* < 0.005).

For potato samples immersed in water, volume changes followed the same trend as mass changes. The greatest volume increase occurred at the highest temperature (−3 °C) due to the increase in turgor cell and swelling of the cellular components as water penetrated and diffused inside the cellular tissue. At lower freezing temperatures, the increase in volume due to swelling was counteracted by the decrease in volume due to more cellular damage at higher pressures. Therefore, the overall volume changes depended on the balance of these two effects. For vacuum-packed potato samples, volume losses were higher at lower temperatures due to more cell lysis from the increased pressure. Potato samples immersed in AA solution did not experience significant changes in volume over most of the experimental conditions. There was 5% increase in volume at −3 °C and 0.16 MPa/s due to pressure-induced impregnation and 8.7% decrease in volume at −15 °C and 0.16 MPa/s due to cellular damage. These results further indicated that lower compression rates caused less cellular damage for samples processed in isotonic solution.

### 3.3. Effects of Isochoric Freezing Conditions on Water Holding Capacity (WHC)

The WHC of fresh samples was 92.5 ± 0.6%. The ANOVA analysis of the factorial regression model (R2 = 98.18) showed that the freezing temperature had, by far, the greatest effect on water holding capacity (F3,48 = 831.69, *p* < 0.001), followed by the processing procedure (F2,48 = 16.87, *p* < 0.001).

**Figure 3:**
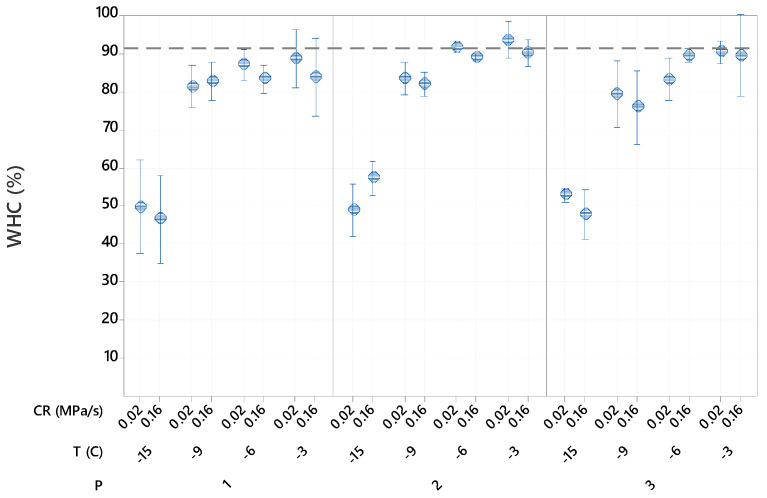
Effects of the freezing procedures (P), freezing temperatures (T) and compression rates (CR) on water holding capacity of cut potatoes after thawing. The pressures were 37 MPa at −3 °C, 71 MPa at −6 °C, 101 MPa at −9 °C and 156 MPa at −15 °C. Data represent mean and 95% confidence interval for the mean.

All isochoric samples had decreased WHC at lower freezing temperatures, as shown in Figure 3. This was due to the concomitant increase in pressure that might have led to an increase in cell membrane permeability from broken cells, allowing liquid release. Prestamo and Arroyo [22] reported similar results and found higher movement of water and metabolites from inside of the cells to outside of the cells when treated with high pressure due to an increase in cell permeability. In general, our results showed significant liquid release at pressures greater than 101 MPa. Dornenburge and Knorr [23] reported an increase in pigment release from plant cell suspensions when they applied pressures greater than 50 MPa.

### 3.4. Effects of Isochoric Freezing Conditions on Color

**Figure 4:**
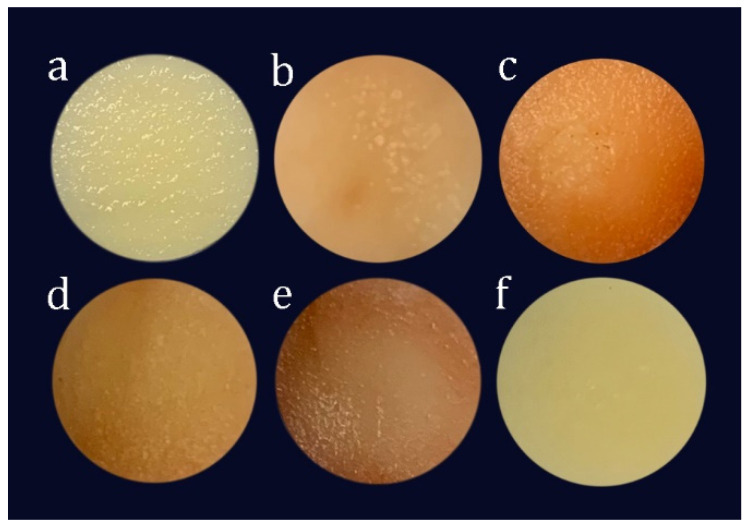
Photographs of (**a**) fresh potato, (**b**) potato sample immersed in water and frozen at 0.02 MPa/s, (**c**) potato sample immersed in water and frozen at 0.16 MPa/s, (**d**) vacuum-packed potato frozen at 0.02 MPa/s, (**e**) vacuum-packed potato frozen at 0.16 MPa/s and (**f**) potato sample immersed in 5% ascorbic acid and frozen at 0.02 MPa/s. The temperature for all the isochoric samples was −15 °C.

Figure 4 shows the appearance of the fresh potato and isochoric frozen potatoes at −15 °C after thawing for one hour. Samples packed in AA solution retained their yellow color independent of the freezing temperature, pressure and compression rate, whereas vacuum-packed samples and those directly immersed in water showed some browning. Additionally, higher compression rates caused greater browning.

**Figure 5:**
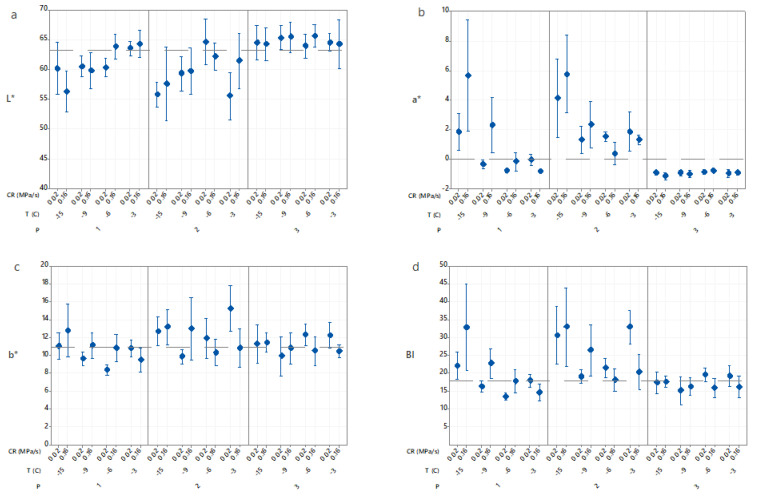
Effects of the freezing procedures (P), freezing temperatures (T) and compression rates (CR) on (**a**) L*; (**b**) a*; (**c**) b* and (**d**) BI values of cut potatoes after thawing. The pressures were 37 MPa at −3 °C, 71 MPa at −6 °C, 101 MPa at −9 °C and 156 MPa at −15 °C. Data represent mean and 95% confidence interval for the mean.

Color parameters (L*, a*, b*) and browning index (BI) values of the samples are shown in Figure 5. The ANOVA analysis of the factorial regression model (R2 = 71.86) showed that the processing procedure (F2,120 = 38.27, *p* < 0.001) and the freezing temperature (F3,120 = 21.64, *p* < 0.001) affected the BI values, whereas compression rate was not a significant factor (Table 1). However, a significant interaction was found between compression rate and processing procedure. Thus, independent of the compression rate, samples immersed in AA retained their color and vacuum-packed samples had higher BI values than samples immersed in water. Additionally, samples immersed in water had significantly lower BI values at the lower compression rate.

Browning occurs when phenolic compounds initially present in the cell vacuoles are oxidized by polyphenol oxidase (PPO), found in the plastids, to o-quinones. O-quinones are highly reactive with amino acids, proteins and phenols, forming an irreversible brown color [24]. For vacuum-packed samples and those immersed in water, BI values increased with a decrease in temperature. Previous authors have also found that isochoric freezing substantially reduced browning in potatoes but does not prevent it [15,16]. This might be due to the destruction of potato tissues and loss of cell compartmentalization at higher pressures and the subsequent increased interactions between polyphenolic substrates and PPO. The increase in BI could also be due to an increase in enzyme activity with pressure. Previous authors had reported an increase in PPO activity with pressure in cut potatoes [15,25]. The vacuum-packed samples also had higher BI values (on average, 41% higher than fresh samples) than for samples immersed in water (on average, 10% higher than fresh samples). This might be due to a lower oxygen level in the water. In addition, the sample in water might have experienced less tissue damage when intercellular spaces were filled with water instead of air due to lower differences in compressibility between cellular components with water than cellular components with air. Moreover, samples immersed in water had larger BI values for higher compression rates, indicating that PPO activity might have been affected by the compression rate in these samples. Potato samples immersed in ascorbic acid had BI values that showed no significant differences with that of fresh potatoes, regardless of freezing temperature or compression rate. Additionally, temperature and compression rate had no significant effect (*p* > 0.05) on their b* values (yellow color) (Figure 5c). Previous authors reported that ascorbic acid markedly suppressed potato PPO activity [26,27]. Ascorbic acid inactivates the PPO enzyme by reducing o-quinones to o-phenolic compounds, thereby preventing enzymatic browning. It also inhibits browning reactions by reducing the oxygen level in the environment [28].

### 3.5. Effects of Isochoric Freezing Conditions on Texture

**Figure 6:**
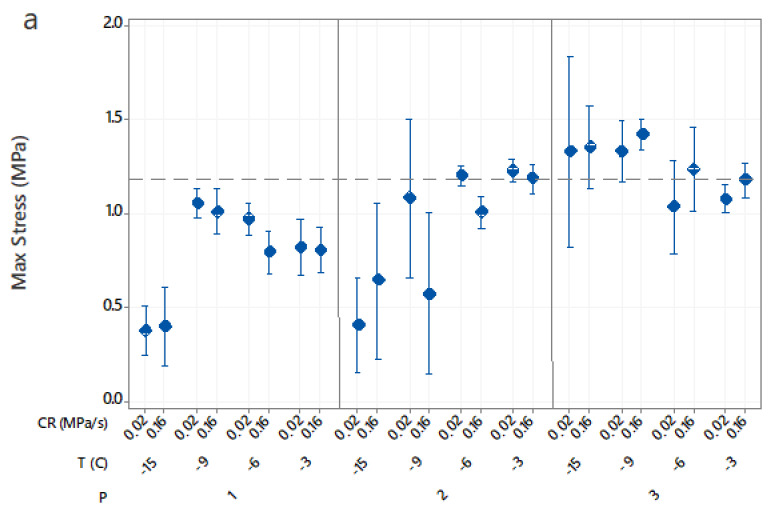

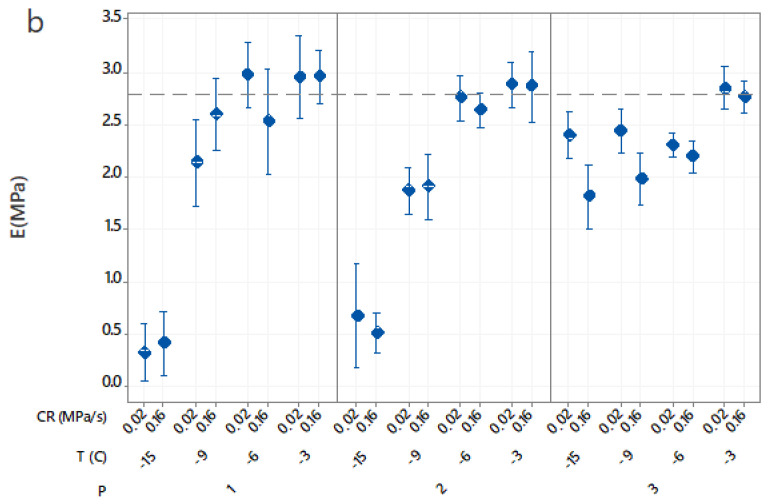
Effects of the freezing variables, freezing procedures (P), freezing temperatures (T) and compression rates (CR) on maximum stress (**a**) and compressive modulus (**b**) of cut potatoes after thawing. The pressures were 37 MPa at −3 °C, 71 MPa at −6 °C, 101 MPa at −9 °C and 156 MPa at −15 °C. Data represent mean and 95% confidence interval for the mean.

The maximum stress and elasticity modulus values for the isochoric samples are shown in Figure 6. The fresh sample had a maximum stress value of 1.18 ± 0.16 MPa and an elasticity modulus (E) value of 2.83 ± 0.08 MPa. For processed samples, the maximum stress values ranged from 20% increase to 68% decrease and the elasticity modulus values ranged from 5% increase to 89% decrease, depending on the isochoric freezing conditions. From the ANOVA of the factorial regression model (R2 = 89.83% for max stress and R2 = 96.16% for elasticity modulus), the procedure was the most significant factor (F2,72 = 117.20, *p* < 0.001) followed by the freezing temperature (F3,72 = 34.62, *p* < 0.001) for the maximum stress values. In comparison, freezing temperature was the most significant factor (F3,72 = 389.16, *p* < 0.001) followed by procedure (F2,72 = 22.74, *p* < 0.001) for the elasticity modulus values. The compression rate did not significantly affect the maximum stress values and was the least significant factor for the elasticity modulus values (Table 1).

Samples directly immersed in water showed some softening at −3 °C due to the hydration of cell wall components, whereas more severe texture loss occurred at −15 °C. Knorr [29] showed that high pressures caused the rupture of cellular membranes, leading to a loss in turgor and permanent damage to the texture. Other authors had also shown that high pressures physically disrupted the cell wall structure of vegetables during pressurization, allowing contact between substrate and hydrolytic enzymes, which accelerated the enzymatic lysis of vegetable tissues [30,31,32,33]. Vacuum-packed samples had the most textural degradation with pressure since severe texture loss occurred at −9 °C. In comparison, samples immersed in AA solution had comparable texture properties to those of the fresh sample, although samples frozen at −9 °C or −15 °C were harder (higher maximum force) than the fresh sample. Previous studies had reported an increase in firmness with high pressure treatments in pressure-shift frozen potato tissues [34] as well as other high-pressure-treated plant tissues [30,35]. Stute et al. [36] hypothesized that the hardening of the tissue was due to insufficient inactivation of the pectin methyl esterase (PME) enzyme during high pressure application. In the intact cell, PME is bound to the cell wall. PME is liberated upon high pressure treatment and contacts highly methylated pectin, which causes desterification. The de-esterified pectin (low methoxy pectin) can form a gel-network with divalent ions like Ca and Mg, contributing to the improved hardness value.

## 4. Conclusions

Effects of isochoric freezing variables (processing procedure, freezing temperature/pressure and compression rate) on the quality properties (mass change, volume change, water holding capacity, color and texture) of thawed cut potatoes were investigated. Results showed that the freezing temperature/pressure and the processing procedure had the greatest effects on the quality of thawed potatoes. Samples treated at high subfreezing temperatures with pressures below 71 MPa retained their overall quality, independent of the processing procedure. The samples showed increased losses in mass, volume, water holding capacity and texture at higher pressures. However, samples immersed in an isotonic solution of 5% ascorbic acid showed improved quality retention. Compression rate had a greater effect for these samples, with better quality retention at lower compression rates. These results show that isochoric freezing could be effectively used to preserve raw cut potatoes in the unfrozen state at subfreezing temperatures. Moreover, isochoric freezing does not require any alterations to current refrigeration infrastructure since isochoric chambers may be stored in any industrial freezer. Thus, isochoric freezing may be regarded as a value-added process within the current food cold chain. However, there is still a need to design and manufacture industrial-scale isochoric freezing chambers to cost-effectively meet industrial needs.

## Figures and Tables

**Table 1:** *F*- and *p*-values for the effects of the factors (processing procedure, freezing temperature/pressure and compression rate) on quality attributes of thawed potatoes frozen under isochoric conditions.

	Mass Change		Volume Change		WHC		BI		Max Stress		Elasticity Modulus	
Parameters	*F*-Value	*p*-Value	*F*-Value	*p*-Value	*F*-Value	*p*-Value	*F*-Value	*p*-Value	*F*-Value	*p*-Value	*F*-Value	*p*-Value
Procedure (P)	379.06	<0.001	719.62	<0.001	16.87	<0.001	38.27	<0.001	117.20	<0.001	22.74	<0.001
Temperature (T)	392.98	<0.001	169.77	<0.001	831.69	<0.001	21.64	<0.001	34.62	<0.001	389.16	<0.001
Compression rate (CR)	6.89	0.010	11.92	0.001	2.93	0.094	0.52	0.474	0.94	0.337	7.88	0.006
P × T	71.86	<0.001	45.61	<0.001	2.62	0.028	6.95	<0.001	27.35	<0.001	57.54	<0.001
P × CR	0.72	0.490	4.15	0.019	1.73	0.188	7.54	0.001	6.86	0.002	6.13	0.004
T × CR	1.72	0.169	12.93	<0.001	1.29	0.289	11.90	<0.001	4.01	0.011	2.27	0.087
P × T × CR	8.75	<0.001	15.80	<0.001	6.19	<0.001	2.04	0.067	4.36	0.001	4.40	0.001

WHC: Water Holding Capacity; BI: Browning Index.

## Data Availability

The data presented in this study are available on request from the corresponding author. The data are not publicly available due to patent restrictions.
